# Rapamycin upregulates glutamate transporter and IL-6 expression in astrocytes in a mouse model of Parkinson's disease

**DOI:** 10.1038/cddis.2016.491

**Published:** 2017-02-09

**Authors:** Yunlong Zhang, Xiaoliang He, Xiaojuan Wu, Ming Lei, Zhiyun Wei, Xiuping Zhang, Lei Wen, Pingyi Xu, Shaomin Li, Shaogang Qu

**Affiliations:** 1 Department of Neurology, The First People's Hospital of Shunde Affiliated to Southern Medical University, Foshan, Guangdong 528300, China; 2 Department of Immunology, School of Basic Medical Sciences, Southern Medical University, Guangzhou, Guangdong 510515, China; 3 Department of Traditional Chinese Medicine, College of Medicine, Xiamen University, Xiamen Fujian 361102, China; 4 Ann Romney Center for Neurologic Diseases, Brigham and Women's Hospital and Harvard Medical School, Boston, MA 02115, USA; 5 Teaching Center of Experimental Medicine, School of Basic Medical Sciences, Southern Medical University, Guangzhou Guangdong 510080, China; 6 Department of Neurology, The First Affiliated Hospital of Guangzhou Medical University, Guangzhou Guangdong 510080, China

## Abstract

Rapamycin protects mice against 1-methyl-4-phenyl-1,2,3,6-tetrahydropyridine (MPTP)-induced loss of dopaminergic neurons, which is an established model for Parkinson's disease. We demonstrated that rapamycin preserves astrocytic expression of glutamate transporters and glutamate reuptake. The protective effect was also observed in astrocyte cultures, indicating that rapamycin acts directly on astrocytes. In the MPTP model, rapamycin caused reduced expression of the E3 ubiquitin ligase Nedd4-2 (neuronal precursor cell expressed developmentally downregulated 4-2) and reduced colocalization of glutamate transporters with ubiquitin. Rapamycin increased interleukin-6 (IL-6) expression, which was associated with reduced expression of inflammatory cytokines, indicating anti-inflammatory properties of IL-6 in the MPTP model. NF-*κ*B was shown to be a key mediator for rapamycin, whereas Janus kinase 2, signal transducer and activator of transcription 3, phosphoinositide 3-kinase, and Akt partially mediated rapamycin effects in astrocytes. These results demonstrate for the first time in a Parkinson's disease animal model that the neuroprotective effects of rapamycin are associated with glial and anti-inflammatory effects.

The motor symptoms of Parkinson's disease (PD) are predominantly due to the degeneration of dopaminergic (DA) neurons in the pars compacta of substantia nigra (SNpc),^1^ and multiple mechanisms are believed to contribute to neurodegeneration in PD, including *α*-synuclein toxicity,^2^ mitochondrial impairment,^3^ reactive oxygen species,^4^ glutamate excitotoxicity,^5, 6, 7^ and increased inflammation.^8, 9^ The 1-methyl-4-phenyl-1,2,3,6-tetrahydropyridine (MPTP) of PD model recapitulates the motor symptoms and preferential cell loss in the SNpc. Its features also include mitochondrial dysfunction, glutamate excitotoxicity, inflammatory response, and so on*.*^10, 11^

Rapamycin inhibits the serine/threonine kinase mTOR (mammalian target of rapamycin) within the mTOR complex 1, resulting in increased autophagy, and in mice increased lifespan.^12^ Rapamycin has also been verified to be neuroprotective in both 6OH-dopamine and MPTP models of PD, and shows different effects compared with the nonselective mTOR inhibitor torin, because of complete and partial inhibition of mTOR.^13^ A previous study reveals that RTP801(REDD1; Ddit4) is a proapoptotic protein that is sufficient and necessary to induce neuronal death in cellular and animal models of PD, and rapamycin blocks increased expression of RTP801 in DA neurons and preserves phosphorylation of phosphoinositide 3-kinase at a critical residue.^13^ However, the effects of rapamycin on glia have not been well characterized in animal models of PD.

In this study, we demonstrate that rapamycin directly targets glial cells to limit two well-established contributing factors to PD pathology: astrocyte activation and the inflammatory response. The effects of rapamycin may be mediated via ubiquitin/proteasomal pathways and the modulation of the Janus kinase 2 (JAK2)/signal transducer and activator of transcription 3 (STAT3) pathway. These findings expand our understanding of the protective mechanisms of rapamycin in a PD model and provide additional potential targets for the therapeutic intervention of PD.

## Results

### Rapamycin is neuroprotective in the MPTP model of PD

**Figure 1:**
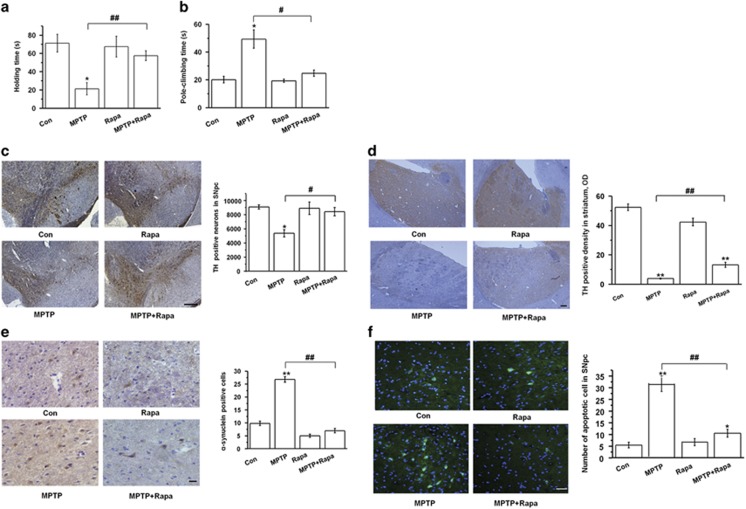
Neuroprotective effects of rapamycin in MPTP-treated mice. (**a** and **b**) Behavioral test results of MPTP-treated mice. (**a**) Grasping test and (**b**) Pole-climbing test (*n*=12 per group, one-way analysis variance (ANOVA)). (**c** and **d**) IHC of TH in the SNpc (panel c) and striatum (panel d) (*n*=6 per group, one-way ANOVA). Scale bar, 100 *μ*m. (**e**) IHC of *α*-synuclein in the SNpc (*n*=6 per group, one-way ANOVA). Scale bar, 40 *μ*m. (**f**) TUNEL staining of apoptotic neurons in the SNpc (*n*=6 per group, one-way ANOVA). Scale bar, 50 *μ*m. Each bar represents the mean±S.E.M. of at least three independent experiments. *P<0.05, **P<0.01, compared with the control group; ^#^*P*<0.05, ^##^*P*<0.01, for the MPTP *versu*s MPTP+Rapa groups

Rapamycin has been shown to protect against neuron death in *in vitro* and *in vivo* models of PD.^13^ To verify its protective effects in MPTP model, we assigned mice to four treatment groups: saline+vehicle (Con), saline+rapamycin (Rapa), MPTP+vehicle (MPTP), and MPTP+rapamycin (MPTP+Rapa). Compared with the Con group, MPTP model displayed typical behavioral and histopathological deficits, including reduced holding time, increased climbing time, and marked reduction of tyrosine hydroxylase (TH) expression in the SNpc and striatum (Figures 1a–d). Treatment with Rapa had no effect compared with Con treatment. However, MPTP+Rapa treatment led to a significant reversal of the effects of MPTP on holding time (*P*<0.01, panel a), climbing time (*P*<0.05, panel b), and TH expression in the SNpc (*P*<0.05, panels c) and striatum (*P*<0.01, panels d). Rapamycin also significantly prevented MPTP-induced *α*-synuclein expression in the SNpc (*P*<0.01, Figure 1e) and prevented MPTP-induced increase in TUNEL-positive cells in the SNpc, indicating prevention of MPTP-induced nigral cell death (Figure 1f). These results verify the protective effects of rapamycin in the MPTP mouse model of PD.

### Rapamycin increases the expression and function of glutamate transporters in *in vitro* and *in vivo* models of PD

**Figure 2:**
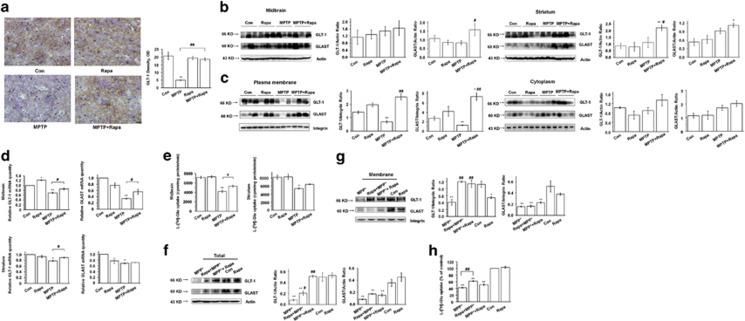
Rapamycin increases the expression and function of GLTs in MPP^+^-treated astrocytes and in MPTP-treated mice. (**a**) IHC of GLT-1 expression in the SNpc (*n*=6 per group, one-way analysis variance (ANOVA)). Scale bar, 40 *μ*m. (**b**) Western blotting of GLTs in total protein lysates from the midbrain and striatum (*n*=3 per group, one-way ANOVA). (**c**) Western blotting of GLTs in membrane and cytoplasmic preparations from the midbrain and striatum (*n*=3 per group, one-way ANOVA). (**d**) qRT-PCR of GLT mRNA expression in the midbrain and striatum (*n*=6 per group, one-way ANOVA). (**e**) Glutamate uptake of synaptosomes from the midbrain and striatum (*n*=6 per group, one-way ANOVA). (**f** and **g**) Western blotting of GLTs in total and membrane protein preparations of astrocytes (at least three experiments, one-way ANOVA). (**h**) Effects of MPP^+^ and rapamycin on glutamate uptake in astrocytes (at least three experiments in triplicate, one-way ANOVA). Each bar represents the mean±S.E.M. of at least three independent experiments. *P<0.05, **P<0.01 compared with the control group; ^#^*P*<0.05, ^##^*P*<0.01, for MPTP *versus* MPTP+Rapa and for MPP^+^ *versus* Rapa+MPP^+^ or MPP^+^+Rapa groups

To test if the protective effect of rapamycin in the MPTP model may be explained, in part, by increased glutamate transporter (GLT) expression, antibodies against GLT-1 were used for histology. We showed that MPTP reduced GLT-1 expression in the SNpc, but that reduction was attenuated in the MPTP+Rapa group (*P*<0.01) (Figure 2a). Rapamycin prevented MPTP-induced reduction of glutamate/aspartate transporter (GLAST) expression in the midbrain and GLT-1 expression in the striatum at the total level (*P*<0.05) (Figure 2b). Furthermore, rapamycin-mediated preservation of GLT-1 and GLAST in MPTP-treated mice was apparent in plasma membrane-enriched but not in the cytoplasmic protein fractions from the midbrain (*P*<0.01) (Figure 2c). The effects of MPTP and rapamycin on the GLTs in MPTP model were confirmed by quantitative real-time PCR (qRT-PCR) (Figure 2d). Within MPTP decreased GLT-1 and GLAST mRNA expression in the midbrain and striatum (0.69±0.03 *versus* 1 for GLT-1 mRNA in the midbrain, *P*<0.01; 0.34±0.02 *versus* 1 for GLAST mRNA in the midbrain, *P*<0.01; 0.78±0.02 *versus* 1 for GLT-1 mRNA in the striatum, *P*<0.05; 0.69±0.02 *versus* 1 for GLAST mRNA in the striatum, *P*<0.05, respectively). While rapamycin rescued the decreased GLT-1 and GLAST mRNA expression in the midbrain and striatum (0.86±0.04 *versus* 0.69±0.03 for GLT-1 mRNA in the midbrain, *P*<0.05; 0.56±0.08 *versus* 0.34±0.02 for GLAST mRNA in the midbrain, *P*<0.05; 0.90±0.02 *versus* 0.78±0.02 for GLT-1 mRNA in the striatum, *P*<0.05, respectively). These results indicate that the expression of GLTs was regulated, at least in part, at the transcriptional level by rapamycin. Moreover, the functional effects of MPTP and rapamycin on GLTs were verified by glutamate uptake assays in the midbrain (*P*<0.01 and *P*<0.05, respectively) (Figure 2e).

GLT-1 and GLAST are expressed by astrocytes in the brain; therefore, we speculated that rapamycin may directly target astrocytes to regulate their expression, rather than acting indirectly by inducing prosurvival effects on neurons. To address this possibility, we treated astrocyte cultures with rapamycin and MPP^+^ (1-methyl-4-phenylpyridinium), the active metabolite of MPTP. To account for potential effects of differential order of addition of the drugs, rapamycin was tested both before and subsequent to MPP^+^ treatment. Western blotting of total and plasma membrane lysates demonstrated that rapamycin prevented MPP^+^-induced decline in GLT-1 expression (*P*<0.01 and *P*<0.05, respectively), but that GLAST expression was not affected by rapamycin cotreatment (*P*>0.05) (Figures 2f and g). The increased GLT-1 expression was functionally relevant, as indicated by the ability of rapamycin to preserve partially the glutamate reuptake capacity of astrocytes cotreated with MPP^+^ (*P*<0.01) (Figure 2h).

We previously reported that GLT dysfunction induced by high glutamate levels impairs hippocampal long-term potentiation (LTP).^14, 15^ Thus, we perform further experiments in the hippocampus to assess the neuroprotective effects of rapamycin on the PD model. GLT-1 and GLAST levels were significantly increased in the hippocampus after rapamycin administration, and rapamycin partially restores the MPTP-induced decrease in GLT-1 (Supplementary Figure S1a). Additionally, GLT-1 and GLAST mRNA expression in the hippocampus was decreased in MPTP-treated mice and partially restored in MPTP+Rapa-treated mice (Supplementary Figures S1b and S1c). We then examined the effects of MPTP on hippocampal synaptic plasticity in acute brain slices. In contrast to the results of a previous study in which 25 *μ*M MPTP increased the baseline in an unsubmerged recording chamber,^16^ we demonstrated that 25 *μ*M MPTP did not increase the baseline and instead partially blocked the LTP in a submerged chamber (MPTP: 132±4%, *n*=6 *versus* vehicle: 158±6%, *n*=8, *P*<0.01) (Supplementary Figure S1d). Interestingly, 200 nM rapamycin alone had no effect on the LTP (152±7%, *n*=8), but this low dose of rapamycin could prevent the effects of MPTP on the LTP (160±9%, *n*=8) (Supplementary Figure S1e). Thus, rapamycin also modulates GLT expression in the hippocampus and prevents MPTP-induced synaptotoxicity.

### Rapamycin decreases the ubiquitination of GLTs in the MPTP model

**Figure 3:**
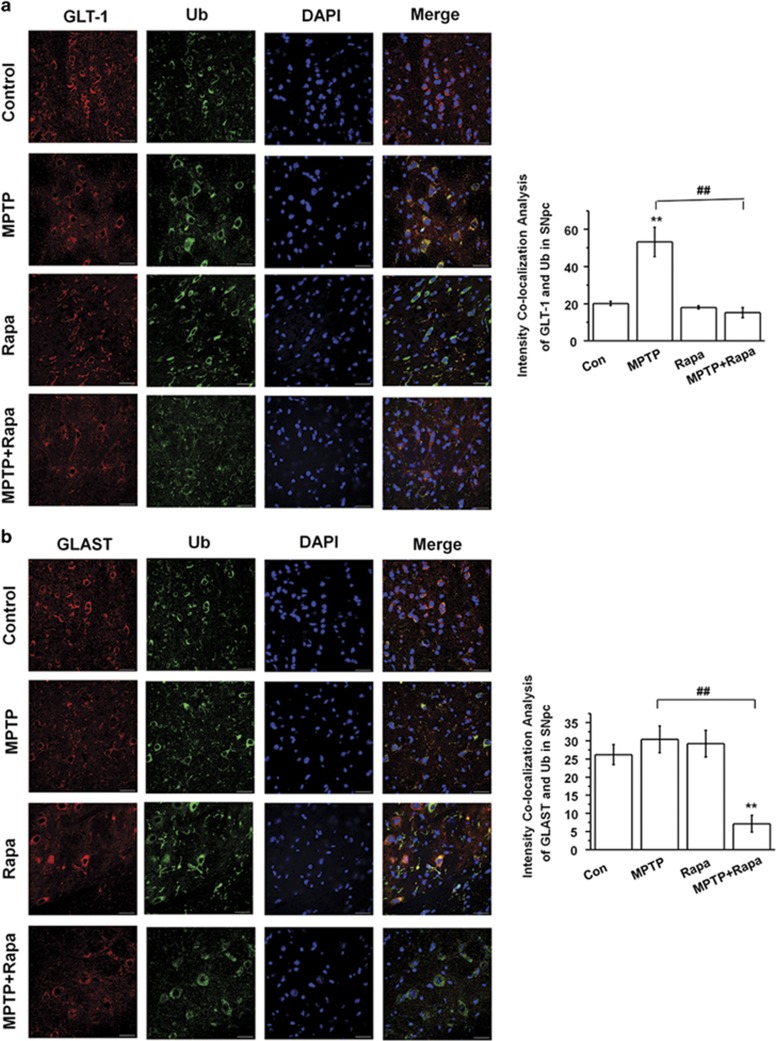
Rapamycin decreases the ubiquitination of GLTs in MPTP-treated mice. (**a** and **b**) Colocalization of ubiquitin and GLTs in the SNpc was assessed by immunofluorescence (*n*=6 per group, one-way analysis variance (ANOVA)). Scale bar, 30 *μ*m. Each bar represents the mean±S.E.M. of at least three independent experiments. **P<0.01, compared with the control group; ^##^*P*<0.01 for MPTP *versus* MPTP+Rapa groups

To explore ubiquitination as a potential mechanism contributing to the rapamycin-induced preservation of GLT, we performed immunofluorescence double staining assays for SNpc sections. MPTP increased the colocalization of GLT-1 with ubiquitin, but this colocalization was reversed by MPTP+Rapa treatment (Figure 3a). Interestingly, MPTP+Rapa treatment also reduced GLAST colocalization with ubiquitin, although neither compound alone had a significant effect on colocalization (Figure 3b). These findings suggest that rapamycin can inhibit MPTP-induced ubiquitination of GLT-1, and potentially GLAST.

**Figure 4:**
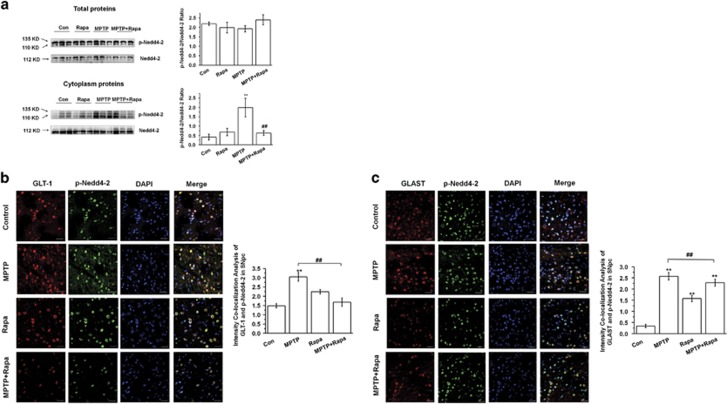
Decreased ubiquitination of GLTs in the treatment of rapamycin is mediated by Nedd4-2 in MPTP-treated mice. (**a**) Western blotting of phospho (p)-Nedd4-2 and Nedd4-2 in total and cytoplasmic protein preparations (*n*=3 per group, one-way analysis variance (ANOVA)). (**b** and **c**) Colocalization of p-Nedd4-2 with GLTs was determined by immunofluorescence (*n*=6 per group, one-way ANOVA). Scale bar, 30 *μ*m. Each bar represents the mean±S.E.M. of at least three independent experiments. **P<0.01, compared with the control group; ^##^*P*<0.01 for MPTP *versus* MPTP+Rapa groups

The E3 ubiquitin ligase Nedd4-2 (neuronal precursor cell expressed developmentally downregulated 4-2) has been implicated in the regulation of astroglial glutamine transporters after manganese treatment.^17^ Therefore, we assessed whether MPTP and rapamycin regulates Nedd4-2 activity. We show that MPTP increased the cytoplasmic expression of phosphorylated Nedd4-2 and its colocalization with GLT-1 as assessed by both immunofluorescence staining and western blotting (*P*<0.01). Furthermore, the increase was prevented by rapamycin cotreatment (*P*<0.01) (Figures 4a and b). MPTP also led to increased colocalization of GLAST and Nedd4-2 (*P*<0.01), whereas rapamycin significantly reduced this colocalization (*P*<0.01) (Figure 4c). Accordingly, we speculate that rapamycin may reverse the effects of MPTP on GLTs by inhibiting Nedd4-2-dependent ubiquitination.

### Rapamycin promotes IL-6 secretion and expression, and activates the JAK2/STAT3 pathway in MPP^+^-treated astrocytes

**Figure 5:**
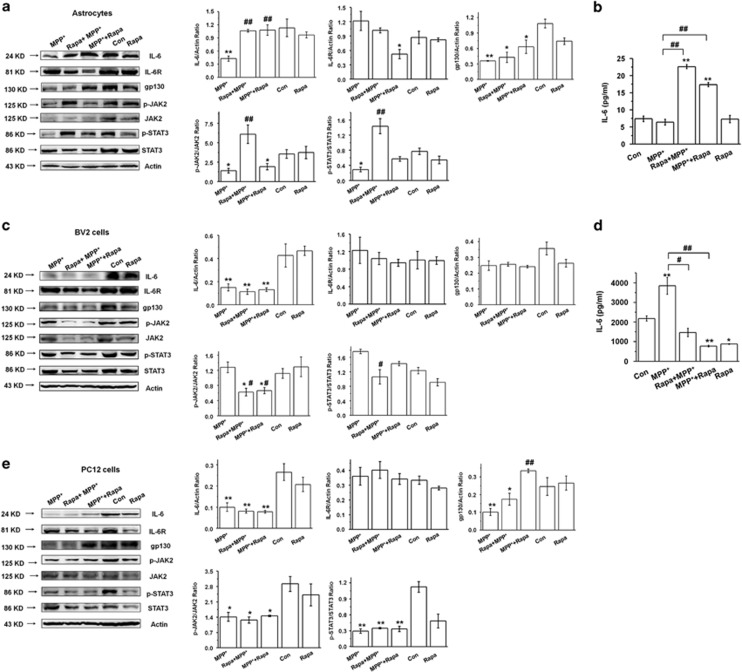
Rapamycin promotes astrocyte-derived IL-6 secretion, increased IL-6 expression and downstream activation of JAK2/STAT3 in MPP^+^-treated astrocytes. (**a**) Western blotting of the IL-6/JAK2/STAT3 pathway, IL-6R and gp130 (at least three experiments, one-way analysis variance (ANOVA)). (**b**) ELISA of IL-6 expression in the astrocyte culture supernatant (at least three experiments in triplicate, one-way ANOVA). (**c**) Western blotting of IL-6/JAK2/STAT3, IL-6R, and gp130 in BV2 cells (at least three experiments, one-way ANOVA). (**d**) ELISA of IL-6 expression in microglial BV2 cell culture supernatants (at least three experiments in triplicate, one-way ANOVA). (**e**) Western blotting of the expression of IL-6/JAK2/STAT3 pathway, IL-6R and gp130 following application of rapamycin in MPP^+^-treated PC12 cells (at least three experiments, one-way ANOVA). Each bar represents the mean±S.E.M. of at least three independent experiments. *P<0.05, **P<0.01, compared with the control group; ^#^*P*<0.05, ^##^*P*<0.01, for MPP^+^ *versus* Rapa+MPP^+^ or MPP^+^+Rapa groups

Interleukin (IL-6) is a major immunomodulatory cytokine with both pro- and anti-inflammatory activities in the pathogenesis of neurodegenerative diseases.^18, 19, 20, 21^ It has also been reported to have protective effects *in vitro* against MPP^+^-induced neurotoxicity.^22^ Therefore, we investigated the effect of rapamycin on IL-6 in MPP^+^-treated astrocytes. In both pre- and postrapamycin treatment paradigms, the combination of rapamycin and MPP^+^ over MPP^+^ alone induced significantly greater IL-6 expression and secretion (Figures 5a and b, *P*<0.01). This elevated IL-6 expression was accompanied by a decreased IL-6R expression, which potentially could be a compensatory mechanism (*P*<0.05) (Figure 5a, second row). Moreover, pre-treatment with rapamycin before MPP^+^ increased the phosphorylation levels of JAK2 and STAT3 (*P*<0.01), which indicates activation of the JAK2/STAT3 signaling cascade. The effect was significant in the pre-treatment paradigm but was less robust and did not reach statistical significance in the post-treatment paradigm (Figure 5a, last 5 rows). IL-6 expression and activation of JAK2/STAT3 pathway were not detected when rapamycin was applied to astrocytes after 48 h of treatment with MPP^+^ (Supplementary Figure S2a). Thus, in subsequent experiments, we treated astrocytes with MPP^+^ for 24 h.

IL-6 secreted from microglia contributes to the inflammatory reaction in PD.^23, 24, 25^ Here we found that rapamycin did not significantly change the expression of IL-6, IL-6R, or gp130 in MPP^+^-treated microglial BV2 cells (*P*>0.05) (Figure 5c, first 3 rows). Furthermore, consistent with previous evidence,^26^ MPP^+^ increased the secretion of IL-6 in BV2 cells, and this increase was blocked by rapamycin in both pre- and post-treatment paradigms (*P*<0.05 and *P*<0.01, respectively) (Figure 5d). Rapamycin also decreased the expression of phospho-JAK2 and phospho-STAT3 in MPP^+^-treated microglial BV2 cells (*P*<0.05) (Figure 5c, last 5 rows). This suggests that, unlike in astrocytes, in MPP^+^-treated microglial BV2 cells rapamycin suppresses IL-6 and the JAK2/STAT3 pathway.

A previous study reported an MPP^+^-induced decrease of IL-6 mRNA expression in PC12 cells (a cellular model of DA neurons), thereby contributing to MPP^+^-induced cell death.^27^ Here we confirm that IL-6 expression is decreased in MPP^+^-treated PC12 cells (*P*<0.01). Additionally, the general phosphorylation status of JAK2/STAT3 pathway was low, and rapamycin had no obvious effects on JAK2/STAT3 phosphorylation in MPP^+^-treated PC12 cells (*P*>0.05) (Figure 5e). These findings suggest that activation of IL-6/JAK2/STAT3 pathway upon rapamycin/MPP^+^ combination treatment may be limited to astrocytes.

### Rapamycin promotes astrocyte-derived IL-6 expression in the MPTP model

**Figure 6:**
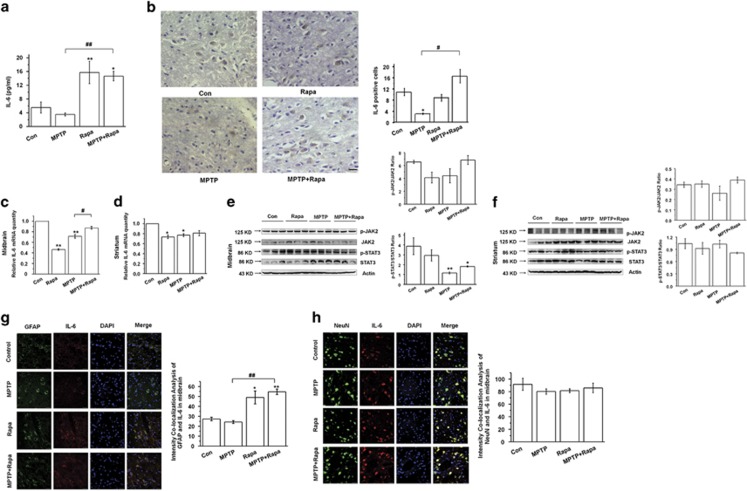
Rapamycin promotes IL-6 secretion in the serum and increases IL-6 expression in the midbrain of MPTP-treated mice. (**a**) ELISA of serum IL-6 levels (*n*=6 per group, one-way analysis variance (ANOVA)). (**b**) IHC of IL-6 expression in the SNpc (*n*=6 per group, one-way ANOVA). Scale bar, 40 *μ*m. (**c** and **d**) qRT-PCR of IL-6 mRNA expression in the midbrain and striatum (*n*=6 per group, one-way ANOVA). (**e** and **f**) Western blotting of JAK2/STAT3 expression in the midbrain and striatum (*n*=3 per group, one-way ANOVA). (**g** and **h**) Immunofluorescence of IL-6 expression in astrocytes or neurons of the SNpc (*n*=6 per group, one-way ANOVA). Scale bar, 30 *μ*m. Each bar represents the mean±S.E.M. of at least three independent experiments. *P<0.05, **P<0.01, compared with the control group; ^#^*P*<0.05, ^##^*P*<0.01, for MPTP *versus* MPTP+Rapa group

We further explored whether this phenomenon can be replicated in MPTP-treated mice. Rapamycin was found to significantly increase IL-6 concentrations in the serum of control and MPTP-treated mice (*P*<0.01) (Figure 6a). IL-6 protein and mRNA expression were also increased in MPTP+Rapa *versus* MPTP-treated mice in the SNpc as assessed by immunohistochemistry (IHC) (Figure 6b, *P*<0.05) and in the midbrain as assessed by qRT-PCR (0.88±0.03 *versus* 0.72±0.04, *P*<0.05) (Figure 6c), but this increase was not observed in the striatum (*P*>0.05) (Figure 6d). However, rapamycin did not affect the phosphorylation status of JAK2/STAT3 pathway in the midbrain or striatum of MPTP-treated mice (*P*>0.05) (Figures 6e and f).

To examine the cellular source of increased IL-6 levels in the CNS, we found that rapamycin increased IL-6 expression specifically in astrocytes in Rapa *versus* Con mice and MPTP+Rapa *versus* MPTP mice (*P*<0.05 and *P*<0.01, respectively) (Figure 6g), but rapamycin had no significant effect on neuronal IL-6 expression (*P*>0.05) (Figure 6h). These results verify the *in vivo* effects of rapamycin on IL-6 in MPTP-treated mice and support *in vitro* results suggesting that the effects occur at the level of astrocytes.

### Rapamycin upregulates IL-6 and GLTs via the mTOR-Akt-NF-*κ*B cascade

**Figure 7:**
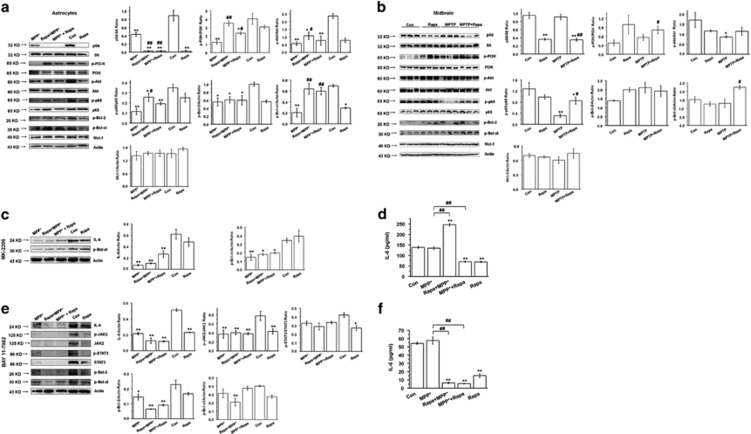
Rapamycin upregulates the IL-6/JAK2/STAT3/Bcl-xl pathway via the mTOR-Akt-NF-*κ*B cascade. (**a** and **b**) Western blotting of a panel of signal molecules in MPP^+^-treated astrocytes and in MPTP-treated mice (at least three experiments, one-way analysis of variance (ANOVA)). (**c**) The effect of MK-2206 on the expression of IL-6 and phospho (p)-Bcl-xl was determined by western blotting (at least three experiments, one-way ANOVA). (**d**) The effect of MK-2206 on IL-6 secretion in cell culture supernatant was determined by ELISA (at least three experiments in triplicate, one-way ANOVA). (**e**) The effect of BAY 11-7082 on the expression of IL-6/JAK2/STAT3, p-Bcl-2, and p-Bcl-xl was determined by western blotting (at least three experiments, one-way ANOVA). (**f**) The effect of BAY 11-7082 on IL-6 secretion in culture supernatants was determined by ELISA (at least three experiments in triplicate, one-way ANOVA). Each bar represents the mean±S.E.M. of at least three independent experiments. *P<0.05, **P<0.01, compared with the control group; and ^#^*P*<0.05, ^##^*P*<0.01, for MPP^+^ *versus* Rapa+MPP^+^ or MPP^+^+Rapa

**Figure 8:**
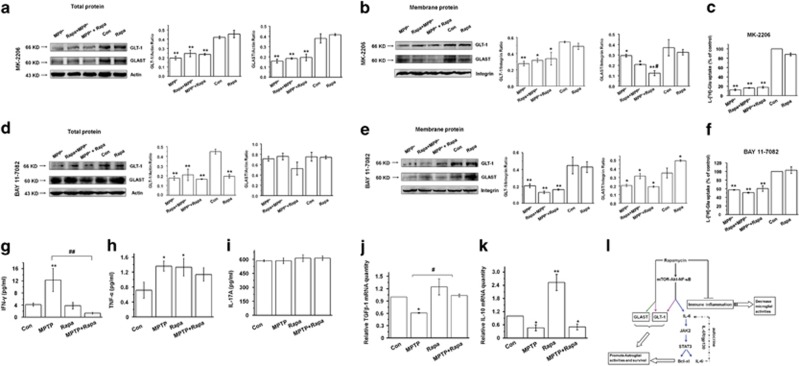
Rapamycin upregulates GLTs via the mTOR-Akt-NF-*κ*B cascade in MPP^+^-treated astrocytes and attenuates immune inflammation in MPTP-treated mice. (**a** and **b**) The effect of MK-2206 on the expression of GLT-1 and GLAST in total and membrane protein preparations was determined by western blotting (at least three experiments, one-way analysis of variance (ANOVA)). (**c**) The effect of MK-2206 on glutamate uptake in MPP^+^-treated astrocytes (at least three experiments in triplicate, one-way ANOVA). (**d** and **e**) The effect of BAY 11-7082 on GLT-1 and GLAST expression at the total and membrane level was determined by western blotting (at least three experiments, one-way ANOVA). (**f**) The effect of BAY 11-7082 on the glutamate uptake in MPP^+^-treated astrocytes (at least three experiments in triplicate, one-way ANOVA). Each bar represents the mean±S.E.M. of at least three independent experiments. *P<0.05, **P<0.01, compared with the control group; and ^#^*P*<0.05, for MPP^+^ *versus* MPP^+^+Rapa. (**g**–**i**) ELISA of IFN-*γ*, TNF-*α*, and IL-17A levels in the serum of MPTP-treated mice (*n*=6 per group, one-way ANOVA). (**j** and **k**) qRT-PCR of TGF-*β* and IL-10 mRNA in MPTP-treated mice (*n*=6 per group, one-way ANOVA). Each bar represents the mean±S.E.M. of at least three independent experiments. *P<0.05, **P<0.01, compared with the control group; ^#^*P*<0.05, ^##^*P*<0.01, for the MPTP *versus* MPTP+Rapa group. (**l**) Schematic model showing the neuroprotective mechanism of rapamycin in the PD models. Rapamycin upregulates GLTs via the mTOR-Akt-NF-*κ*B cascade, in part, by decreasing the activity of Nedd4-2. Rapamycin also activates astroglial IL-6 signaling via the JAK2/STAT3/Bcl-xl pathway, although the expression of IL-6R is moderated via autocrine mechanisms. Through upregulation of GLTs and the IL-6/JAK2/STAT3 pathway, rapamycin promotes astroglial activities. In addition, rapamycin attenuates immune inflammation in PD models by decreasing microglial activities

Because some effects on IL-6 expression were observed in the absence of regulation of the JAK2/STAT3 pathway, the regulation of additional cell signaling pathways was explored. Rapamycin decreased the *in vitro* and *in vivo* expression of phosphorylated S6, the downstream effector of mTORC1, which confirms that rapamycin engaged its target, mTORC1 (*P*<0.01) (Figures 7a and b, first 2 rows). *In vitro* Rapa+MPP^+^ treatment in astrocytes increased the expression of phosphorylated PI3 kinase (PI3K), Akt, and NF-*κ*B/p65 as compared with MPP^+^ alone (*P*<0.01 and *P*<0.05, respectively) (Figure 7a, last 10 rows), and *in vivo* MPTP+Rapa increased the expression of phosphorylated PI3K and NF-*κ*B/p65 compared with MPTP-only treatment (*P*<0.05) (Figure 7b, last 10 rows), which suggests the possibility that these pathways may regulate IL-6 expression. Therefore, to dissect the pathways regulating IL-6 expression, inhibitors of Akt (MK-2206), NF-*κ*B (BAY 11-7082), and PI3K (LY 294002) were used. MK-2206 application did not significantly affect IL-6 expression in astrocytes treated with rapamycin+MPP^+^, MPP^+^+rapamycin, and MPP^+^ (*P*>0.05) (Figure 7c), and only modestly reduced IL-6 secretion in astrocytes treated with rapamycin±MPP^+^ (*P*<0.01) (Figure 7d). However, BAY 11-7082 robustly blocked the effects of rapamycin on IL-6 protein expression and secretion (Figure 7e, first row and Figure 7f), which suggests that NF-*κ*B is required for rapamycin function. BAY 11-7082 also robustly blocked the phosphorylation of the JAK2/STAT3 pathway (Figure 7e). Furthermore, MK-2206 and BAY 11-7082 eliminated the effects of rapamycin on GLTs expression and function in MPP^+^-treated astrocytes (Figures 8a–f), which suggests that Akt/NF-*κ*B pathway may also regulate the effects of rapamycin on GLT. LY 294002 also decreased IL-6 protein expression in MPP^+^+rapamycin- and MPP^+^-treated astrocytes groups (Supplementary Figure S2c), which suggests that PI3K may also be involved. These results support the possibility that activation of IL-6 by rapamycin in MPP^+^-treated astrocytes (and potentially in MPTP-treated mice) may be regulated predominantly by the mTOR-Akt-NF-*κ*B cascade.

### Rapamycin attenuates immune inflammation in MPTP-treated mice

The effects of rapamycin on other relevant immune cytokines were also tested. Interferon-*γ*(IFN-*γ*) signaling, together with tumor necrosis factor-*α*(TNF-*α*), has an important role in stimulating and maintaining the activation of glial cells in PD.^28, 29^ Also, IL-17 has been shown to induce IL-6 secretion, which further enhances Th17 cell differentiation.^30^ Transforming growth factor-*β*1 (TGF-*β*1) was proved to be a cofactor that potentiates the neurotrophic actions of glial cell line-derived neurotrophic factor (GDNF) in PD animal models.^31^ Besides, AAV2-mediated gene transfer of human IL-10 into the striatum shows neuroprotective role in MPTP model.^32^ Here we found that MPTP treatment led to an increase in serum IFN-*γ* (*P*<0.01) and TNF-*α* (*P*<0.05) (Figures 8g and h), but had no effect on IL-17 levels (Figure 8i, *P*>0.05). MPTP also decreased TGF-*β*1 and IL-10 mRNA expression in the midbrain (0.61± 0.01 *versus* 1, *P*<0.05 in Figure 8j; 0.47±0.14 *versus* 1, *P*<0.05 in Figure 8k). Moreover, rapamycin significantly blocked the MPTP effects on serum IFN-*γ* (*P*<0.01) and midbrain TGF-*β*1 mRNA (1.04±0.04 *versus* 0.61±0.01, *P*<0.05) (Figures 8g and j), but did not influence MPTP-induced changes in TNF-*α* and IL-10 expression (Figures 8h and k, *P*>0.05). These findings indicate that MPTP has proinflammatory effects both in the periphery and in the CNS. A schematic summarizing these findings is shown in Figure 8l.

## Discussion

Glial activation and inflammation are believed to contribute to PD and other aging-related diseases.^33, 34, 35, 36, 37^ Previously, three MPTP models are commonly used because of different MPTP dosing regimens.^38, 39, 40, 41, 42, 43^ Within the subacute MPTP model, the dosing regimens refer to one injection of 20–30 mg/kg MPTP daily for five consecutive days. This model causes striatal dopamine depletion nearly 50% in C57BL/6 mice, and the DA lesion stabilizes by 21 days after MPTP administration.^39, 40^ Besides, in this study, we focus on the expression changes of GLTs in PD and according to our previous work, we find the decreased GLTs, TH expression and movement disorder in different time points in the subacute MPTP model (unpublished data). Thus in this study we chose the subacute MPTP model. In the brain, MPTP is metabolized to 1-methyl-4-phenyl-2,3-dihydropyridinium by the enzyme monoamine oxidase-B within non-DA cells, and then to MPP^+^. Generally, the dose of MPP^+^ used in primary mesencephalic DA neurons is slight, and a significant loss of primary DA neurons cultures is even detected at 0.5 *μ*M MPP^+^.^44, 45^ However, primary astrocytes are less susceptible to MPP^+^ toxicity than DA neurons. According to a previous study and our work, astrocytes show significant loss at the dose of 800 *μ*M MPP^+^.^46, 47^ In this study, we also use the glial glutamate uptake results upon MPP^+^ treatment as the indicator, and thus we chose 1000 *μ*M dose of MPP^+^ as the work concentration.

Rapamycin has been shown to reduce MPTP toxicity;^48^ however, the mechanism has not been well characterized. Here we demonstrated that rapamycin has profound effects on the GLT expression in MPP^+^-treated astrocytes and MPTP-treated mice and prevents MPP^+^/MPTP-induced reduction in glutamate reuptake capacity. While the functional relevance of increased glutamate reuptake capacity has not been formally shown in this study, its relevance is well established in multiple models of PD.^49, 50, 51^ Therefore, it is reasonable to assume that the increase in glutamate reuptake capacity, at least in part, contributes to the neuroprotective effects of rapamycin in the MPTP model. It is well known that glutamate-mediated transmission has a central role in numerous fundamental brain functions, such as synaptic plasticity. Our study is the first to show that rapamycin prevents MPTP-induced hippocampal LTP impairment. This result further supports previous demonstration of cognitive dysfunction in the early phase of MPTP model.^52^ Also, GLT-1 has been demonstrated to be responsible for the increase in glutamate uptake during LTP,^53^ and we found that rapamycin can restore the GLT-1 expression in the hippocampus. Thus, upregulation of GLT-1 upon rapamycin treatment may improve the LTP in MPTP mouse lesions.

We also found that rapamycin reduced the number of astrocytes staining double positive for GLT-1+ubiquitin, GLAST+ubiquitin, GLT-1+phospho-Nedd4-2, and GLAST+phospho-Nedd4-2 in MPTP-treated mice. Consistent with these findings, regulation of GLT via Nedd4-2 has been reported in alternative neurodegenerative disease models.^54, 55, 56^ Our recent unpublished data also suggest that Nedd4-2 knockdown ameliorates movement disorders and increases TH expression in PD mice via decreasing GLT ubiquitination. Thus, in this study we offer a possible role of rapamycin in regulating GLT levels by inhibiting Nedd4-2-dependent ubiquitination in the PD model.

In addition, rapamycin also increased the concentration of IL-6 in the midbrain of MPTP-treated mice and these results were recapitulated in MPP^+^-treated astrocytes. IL-6 can have both proinflammatory and anti-inflammatory effects, and its role in PD remains controversial.^18, 19, 20, 21, 22, 57, 58^ However, MPTP treatment upregulated the proinflammatory markers IFN-*γ* and TNF-*α* and downregulation of the anti-inflammatory markers TGF-*β*1 and IL-10 in the periphery and the CNS, which suggests that MPTP induces a proinflammatory profile. Rapamycin partially reversed the MPTP-induced changes in immune markers, which indicates that the rapamycin-mediated increase in IL-6 levels may be predominantly anti-inflammatory.

We further used pharmacological inhibitors to investigate the cell signaling cascades regulated by rapamycin. Inhibition of NF-*κ*B led to the most robust blockade of rapamycin-mediated regulation of GLT, IL-6, and JAK2/STAT3 phosphorylation. Therefore, activation of NF-*κ*B by rapamycin appears to be a step fairly proximal in the cell signaling cascade that is crucial for mediating the biological effects of rapamycin, whereas PI3K/Akt signaling cascade may be partially responsible for mediating the effects of rapamycin. The findings in this study are also supported by other reports.^59, 60^

Previously, the IL-6/JAK/STAT pathway have been proved to regulate GLTs,^61, 62^ so actually we also want to explore whether the IL-6/JAK/STAT pathway is involved in regulating GLTs in PD. However, here we did not find the connection between the JAK/STAT pathway and GLTs. In our hand, we found that rapamycin increased GLT expression in SNpc (Figure 2c), whereas the JAK/STAT pathway show no obvious change in SNpc (Figure 6e); besides, both pre- and post- treatment of rapamycin increased GLT-1 expression in MPP^+^-treated astrocytes (Figures 2f and g); however, the JAK/STAT pathway only showed significant changes in pre-treatment of rapamycin (Figure 5a). These results suggest that JAK/STAT pathway may share little connection with GLTs in PD, thus we choose to state the neuroprotective effects of IL-6/JAK/STAT pathway in another story. Intriguingly, both GLTs and IL-6 are regulated by the mTOR-Akt-NF-*κ*B cascade, suggesting these two stories are linked to some extent.

In summary, the neuroprotective effects of rapamycin in the MPTP model have for the first time been associated with direct effects of rapamycin on astrocytes that lead to improved glutamate reuptake capacity and, in the presence of elevated IL-6 levels, to a less inflammatory cytokine profile. These findings expand our knowledge about the protective mechanisms of rapamycin in an animal model of PD and provide additional targets for therapeutic interventions in PD.

## Materials and methods

### Reagents

MPP^+^ and MPTP were purchased from Sigma-Aldrich (St. Louis, MO, USA). Dulbecco's modified Eagle's medium/F12 and fetal calf serum were purchased from Hyclone (Logan, UT, USA). Anti-TH and GLT-1 antibodies were purchased from Millipore (Bedford, MA, USA). Anti-GLAST, anti-*α*-synuclein, anti-IL-6, and anti-IL-6 receptor antibodies were purchased from Santa Cruz Biotechnology (Santa Cruz, CA, USA). Anti-phospho-Nedd4-2 (Ser342), anti-Nedd4-2, anti-phospho-S6, and anti-S6 antibodies were purchased from Cell Signaling Technology (Danvers, MA, USA). Anti-phospho-JAK2 (Tyr221), anti-phospho-NF-*κ*B p65 (Ser276), anti-phospho-STAT3 (Tyr705), anti-JAK2, anti-NF-*κ*B p65, anti-STAT3, anti-gp130, anti-phospho-PI3K p85-*α* (Tyr607), anti-PI3K p85-*α*, anti-phospho-Akt (Ser473), anti-Akt, anti-phospho-Bcl-2 (Ser87), and anti-phospho-Bcl-xl (Ser62) antibodies were purchased from EnoGene (Nanjing, China). Rapamycin was purchased from Sangon (Shanghai, China). Actin antibody was purchased from Beyotime (Shanghai, China). PI3K inhibitor (LY 294002), Akt inhibitor (MK-2206), and NF-*κ*B (BAY 11-7082) were purchased from Selleck Chemicals (Houston, TX, USA). Anti-Mcl-1, FITC-conjugated goat anti-mouse, TRITC-conjugated goat anti-rabbit, horseradish peroxidase (HRP)-conjugated goat anti-mouse, and rabbit antibodies were purchased from Boster (Wuhan, China). EZ-Link Sulfo-NHS-SS-Biotin was purchased from Thermo Scientific (Waltham, MA, USA; no. 21331). Trizol was purchased from Invitrogen (Carlsbad, CA, USA). PrimeScript RT Reagent Kits and SYBR Premix Ex Taq Kits were purchased from Takara (Otsu, Japan). IL-6, TNF-*α*, IL-17A, and IFN-*γ* Enzyme-linked Immunosorbent Assay (ELISA) Kits were purchased from BioLegend (San Diego, CA, USA). TUNEL (terminal deoxynucleotidyl transferase-mediated dUTP nick-end labeling) Staining Assay Kits were purchased from Beyotime.

### Animals and cell culture

Ten-week-old, C57BL/6 male mice were obtained from the Guangdong Medical Laboratory Animal Facility (Foshan, China). Primary cortical astrocytes were obtained from newborn C57BL/6 mice as described.^46^ Primary astrocytes, BV2 cells, and PC12 cells were cultured in Dulbecco's modified Eagle's medium/F12 supplemented with 10% fetal calf serum. All animal protocols in this study were approved by the Institutional Animal Care and Use Committee of the Southern Medical University.

### Drug treatment

Stock solutions of MPP^+^ were prepared in 0.01 M PBS and rapamycin were prepared in DMSO. Stock solutions were then diluted in cell culture medium containing half-serum to the appropriate concentrations. The addition of MPP^+^ to astroglial cultures was performed at 1000 *μ*M as stated by our work and others,^46, 63^ and rapamycin was performed at 100 nM as stated by the previous study.^59^ For *in vitro* assays, cells were randomly divided into five groups: (1) *control group*: cells were treated with normal culture medium for 24 h; (2) *MPP^+^ group*: cells were treated with culture medium containing 1000 *μ*M MPP^+^ for 24 h; (3) *Rapa+MPP^+^ group*: cells were pretreated with culture medium containing 100 nM rapamycin for 4 h, rapamycin was removed, and cells were treated with culture medium containing 1000 *μ*M MPP^+^ for 24 h; (4) *MPP^+^+Rapa group*: cells were treated with culture medium containing 1000 *μ*M MPP^+^ for 24 h, MPP^+^ was removed, and cells were treated with culture medium containing 100 nM rapamycin for 4 h; (5) *Rapa group*: cells were treated with culture medium containing 100 nM rapamycin for 4 h, rapamycin was removed, and cells were treated with normal culture medium for 24 h. The control group was treated with PBS or DMSO.

For *in vivo* experiments, mice were randomly divided into four groups: saline+vehicle (Con), MPTP+vehicle (MPTP), saline+rapamycin (Rapa), and MPTP+rapamycin (MPTP+Rapa). According to the previous studies,^38, 64^ MPTP was administered intraperitoneally for 5 consecutive days at a dose of 30 mg/kg free base (MPTP-HCl) in saline. The vehicle for rapamycin was DMSO. Rapamycin or vehicle was administered for 11 consecutive days, starting 2 days before MPTP treatment, at a dose of 7.5 mg/kg, as described.^13^ MPTP/saline injections were performed at 0800 hours and rapamycin/vehicle injections were performed at 2000 hours. One day after the last rapamycin/vehicle injections, behavioral experiments were performed and the animals were killed for tissue collection.

### Behavioral tests

*Grasping test*: Mice were tested with the grasping experiment at different time points, respectively. Briefly, mice were suspended by their forelimbs on a metal rod (diameter: 1.5 mm) and positioned ~30 cm above the box. The holding time on the metal rod was recorded.

*Pole-climbing test*: Mice were placed on the peak of foam ball (diameter: 2.0 cm) fixed on the stick (diameter: 1.0 cm; length: 50 cm). The climb time from the peak to the bottom of the stick was counted

### IHC assay

Brain tissue samples were embedded in optimum cutting temperature compound (Sakura Finetek, Torrance, CA, USA) and stored at −80 °C. Samples sections were cut into 10 *μ*m slices, antigen retrieval was performed using citrate buffer, and endogenous peroxidase activity was blocked with 3% H_2_O_2_ for 10 min at room temperature (RT). Samples were incubated with the indicated primary antibodies overnight at 4 °C. After washing with PBS, slices were incubated with HRP-conjugated secondary antibodies (ZSGB-BIO, Beijing, China) for 1 h at RT. Antibody–peroxidase complexes were revealed by incubating slices with a 3,3-Diaminobenzidine Peroxidase Substrate Kit (Boster, Wuhan, China). The integrated option density was counted at × 400 magnification in at least 10 high-power fields from kidney sections from representative mice in each group using an Image-Pro Plus 6.0 photogram analysis system (IPP 6.0; Media Cybernetics, Bethesda, MD, USA).

### Immunofluorescence

Brain tissue slices were obtained as described for IHC. Slices were fixed in 4% paraformaldehyde, rinsed with PBS, permeabilized with 0.1% Triton X-100, and blocked with 1% BSA. Slices were incubated with primary antibodies overnight at 4 °C, rinsed with PBS, and incubated with FITC-conjugated goat anti-mouse IgG or TRITC-conjugated goat anti-rabbit IgG for 2 h at 37 °C. DAPI was used to stain cell nuclei. Immunostaining was then examined using the Olympus 1 × 81 FV1000 Laser Scanning Confocal Microscope (Shinjuku, Tokyo, Japan). Primary antibodies were replaced with 1% BSA as a negative control.

### Colocalized immunofluorescence assay

Brain tissue slices were fixed in 4% paraformaldehyde and then rinsed with PBS. Then, the slices were permeabilized with 0.1% Triton X-100 and blocked with 5% BSA. For the colocalization study, the slices were incubated with primary antibodies (as indicated in the Figures 3a, b,4b, and 6g) overnight at 4 °C, rinsed with PBS and incubated with FITC-conjugated goat anti-mouse IgG and TRITC-conjugated goat anti-rabbit IgG for 2 h at 37 °C. DAPI was used to stain cell nuclei. Immunostaining was then examined using an Olympus 1 × 81 FV1000 Laser Scanning Confocal Microscope (Shinjuku, Tokyo, Japan). Quantification of double-labeled immunocytological antigens that were colocalized was performed by imaging and analyzing cells using IPP 6.0 (Media Cybernetics, Bethesda, MD, USA). Generally, GLT-1, GLAST, or IL-6 clusters were selected automatically in the pseudocolored ‘red' channel as discrete puncta of intensity >1.5-fold brighter than the background fluorescence. Selected clusters were transferred to the green channel to measure the Ub, phospho-Nedd4-2, GFAP, or NeuN fluorescence. Colocalization of these targeted proteins was measured as the percentage of integrated Ub, phospho-Nedd4-2, GFAP, or NeuN pixel intensities that overlapped with the GLT-1, GLAST, or IL-6 fluorescence in individual clusters. As a negative control, the primary antibody was replaced with 5% BSA.

### TUNEL staining

Apoptotic cells in the SNpc were stained by TUNEL assay according to the manufacturer's instructions (Beyotime). Briefly, brain tissue slices were obtained as described for IHC, and the slices in each group were fixed in 4% paraformaldehyde and rinsed with PBS. Then, the slices were permeabilized with 1.0% Triton X-100 for 5 min and blocked with 5% BSA for 30 min. The fluorescein TUNEL reagent mixture was applied for 60 min at 37 °C. DAPI was used to stain cell nuclei, and then the slices were viewed under a laser scanning confocal microscope.

### qRT-PCR

Total RNA was isolated from brain tissues using Trizol Reagent (Life Technologies, Carlsbad, CA, USA) and cDNA was synthesized using a TaKaRa PrimeScript Reagent Kit with the gDNA Eraser (TaKaRa, Otsu, Japan). The cDNA was then amplified with SYBR Premix Ex Taq (TaKaRa, Otsu, Japan) according to the manufacturer's instructions. Primers used in this study are listed as follows: GLT-1 (forward) 5′-CGATGAGCCAAAGCACCGAA-3′ and (reverse) 5′-CTGGAGATGATAAGAGGGAGGATG-3′; GLAST (forward) 5′-TCAAGTTCTGCCACCCTACC-3′ and (reverse) 5′-TCTGTCCAAAGTTCAGGTCAA-3′; IL-6 (forward) 5′-ACTTCCATCCAGTTGCCTTC-3′ and (reverse) 5′-ATTTCCACGATTTCCCAGAG-3′; TGF-*β*1 (forward) and 5′-ATTCCTGGCGTTACCTTGG-3′ (reverse) 5′-AGCCCTGTATTCCGTCTCCT-3′; IL-10 (forward) 5′-GCAGCCTTGCAGAAAAGAGAG-3′ and (reverse) TCCTGCATTAAGGAGTCGGTT; *β*-actin (forward) 5′-CTACAATGAGCTGCGTGTGGC-3′ and (reverse) CAGGTCCAGACGCAGGATGGC. Fluorescence was detected using a Corbett Research RG-6000 Real-Time PCR Machine (Corbett Life Science, Sydney, NSW, Australia). Each sample was run in triplicate and was compared with actin as the internal control. Results were obtained by using the 2^−ΔΔCT^ method. Briefly, the Ct values of the gene of interest and actin were derived from the software in the real-time PCR machine. ΔCt=Ct(gene of interest)−Ct(actin); ΔΔCt=((Ct gene of interest−Ct actin) rapamycin, PD or rapamycin+PD)−((Ct gene of interest−Ct actin) control); and finally, the 2^−ΔΔCT^ value represents the relative expression of the gene of interest in rapamycin, PD, or rapamycin+PD groups compared with control. Data are from three separate experiments, and each was performed in triplicate.

### Total protein extraction

For the total membrane protein extraction, cells or brain tissues were lysed with radioimmunoprecipitation assay buffer (Beyotime) containing 1 mM PMSF (Beyotime). The protein concentration was measured via BCA Assay Kit (Beyotime, Shanghai, China). Samples were diluted in protein loading buffer, and heated to 95 °C for 5 min.

### Cell surface biotinylation

Cell surface expression levels of GLT-1 and GLAST were tested using the membrane-impermeable biotinylation reagent EZ-Link Sulfo-NHS-SS-Biotin. Cells or tissues were washed two times with ice-cold PBS (pH 8.0) and then incubated with 2.5 ml of EZ-Link Sulfo-NHS-SS-Biotin (0.5 mg/ml in PBS) in two successive 20 min incubations on ice with gentle shaking. Cells or tissues were washed two times with 100 mM glycine to remove non-reacted biotinylation reagent by incubation on ice for 20 min. Then, the cells or homogenized tissues were lysed on ice for 20 min in 750 *μ*l of cell lysis buffer containing the protease inhibitor mixture and 1 mM PMSF. The debris was removed by centrifugation at 12 000 × *g* for 20 min at 4 °C. Supernatants were transferred to new tubes and 200 *μ*l of streptavidin–agarose beads were added to bind the biotin-labeled cell membrane proteins. They were centrifuged at 3 000 × *g* for 1 min at 4 °C and the supernatant was discard, and then were washed three times with ice-cold lysis buffer. At the end, they were washed once with ice-cold PBS and centrifuged at 16 000 × *g* for 1 min at 4 °C. The membrane protein samples were used for western blotting.

### Western blotting assay

Membrane and total protein samples were resolved via 12% SDS-PAGE and transferred to polyvinylidene difluoride (PVDF) membranes. After blocking with 5% BSA for 2 h at RT, the PVDF membranes were incubated with the indicated primary antibodies overnight at 4 °C. The PVDF membranes were incubated with HRP-conjugated secondary antibodies followed by three washes with TBS-T (Tris-buffered saline containing 0.1% Tween-20). Proteins were visualized with enhanced chemiluminescence (Beyotime). Actin immunoreactivity was set as loading control for each protein of interest.

### l-[^3^H]-Glutamic acid uptake assay

l-[^3^H]-Glutamic acid uptake assays were performed as described previously.^46^ Briefly, astrocytes were washed once with choline solution (150 mM choline chloride, 5 mM KP_i_, pH 7.4, 0.5 mM MgSO_4_, and 0.3 mM CaCl_2_), and then 0.4 *μ*Ci per well l-[^3^H]-glutamic acid (specific activity 12.9 Ci/mmol) were added to the wells. The reactions were incubated for 10 min at RT and stopped by two additions of ice-cold NaCl solution (150 mM NaCl, 5 mM KP_i_, pH 7.4, 0.5 mM MgSO_4_, and 0.3 mM CaCl_2_). One percent SDS was added to dissolve cells, and radioactivity was measured by liquid scintillation counting. Data are from three separate experiments, each performed in triplicate.

### Determination of glutamate uptake in the synaptosomes

Midbrain and striatum tissues were homogenized in 0.32 M sucrose solution (0.32 M sucrose, 5 mM HEPES, pH 7.4) and centrifuged at 1000 × *g* for 15 min. The resulting supernatants were centrifuged further at 15 000 × *g* for 30 min, and the pellets were resuspended using 0.32 M sucrose solution to obtain the crude synaptosomes. Protein concentrations were determined by BCA Colorimetric Assay (Beyotime, Shanghai, China). Then, the crude synaptosomes were resuspended in Kreb's buffer (127 mM NaCl, 3.73 mM KCl, 1.8 mM CaCl_2_, 1.18 mM KH_2_PO_4_, 20 mM NaHCO_3_, 2 mM ATP, 2 g/l d-glucose, pH 7.4) in a water bath at 25 °C for 10 min at a final concentration of 0.5 mg/ml. l-[^3^H]-glutamic acid of 1 *μ*Ci was added to the synaptosome preparations in each tube in a water bath at 25 °C for another 10 min, and the reactions were terminated with 5 ml of ice-cold Kreb's buffer. Synaptosomes were washed three times to remove excess labeled glutamate with phosphate-buffered saline and filtrated with 0.22 *μ*m filter paper. The filter paper containing the rinsed synaptosomes were then transferred into scintillation vials containing 3 ml of scintillation cocktail in the dark overnight. The radioactivity was measured by liquid scintillation counting on the next day. The glutamate uptake in the synaptosomes was represented in c.p.m. per mg protein per min. Data are from three separate experiments, each performed in triplicate.

### Enzyme-linked immunosorbent assay (ELISA)

Cytokines in the serum and culture supernatants were measured with ELISA MAX Deluxe Sets (BioLegend) according to the kit instructions. Briefly, each plate was coated with 100 *μ*l of the capture antibody solution and incubated overnight at 4 °C. Plates were washed four times with wash buffer, and 1 × assay diluent solution was added to block nonspecific binding. After incubation for 1 h at RT, sera were added to the wells and incubated for 2 h at RT. Then, plates were incubated with indicated antibodies for 1 h at RT. After four washes, plates were incubated with diluted avidin-HRP solutions for 30 min at RT. After five washes, mixed tetramethylbenzidine substrate was added to each well and the absorbance at 450 nm was measured with a microplate reader (Perkin-Elmer, Waltham, MA, USA). Data were obtained from three separate experiments, each performed in triplicate.

### Hippocampal slice preparations

Mice (C57BL/6x129) were killed by isoflurane anesthesia at age 6–8 weeks for field recordings. Brains were quickly removed and submerged in ice-cold oxygenated sucrose-replaced artificial cerebrospinal fluid (ACSF) cutting solution containing (in mM): 206 sucrose, 2 KCl, 2 MgSO_4_, 1.25 NaH_2_PO_4_, 1 CaCl_2_, 1 MgCl_2_, 26 NaHCO_3_, 10 d-glucose, pH 7.4, 315 mOsm. Transverse slices (350 *μ*m thick) were cut with a vibroslicer from the middle portion of each hippocampus. After dissection, slices were incubated in ACSF containing (in mM): 124 NaCl, 2 KCl, 2 MgSO_4_, 1.25 NaH_2_PO_4_, 2.5 CaCl_2_, 26 NaHCO_3_, 10 d-glucose, pH 7.4, 310 mOsm, in which they were allowed to recover for at least 90 min before recording. A single slice was then transferred to the recording chamber and submerged beneath continuously perfused ACSF saturated with 95% O_2_ and 5% CO_2_. Slices were incubated in this chamber for 20 min before stimulation at RT (~24 °C).

### Electrophysiology

Standard field excitatory postsynaptic potentials (fEPSPs) were recorded in the CA1 region of hippocampus. A bipolar-stimulating electrode (FHC Inc., Bowdoin, ME, USA) was placed in the Schaffer collaterals to deliver test and conditioning stimuli. A borosilicate glass recording electrode filled with ACSF was positioned in the stratum radiatum of CA1, 200–300 *μ*m from the stimulating electrode. fEPSPs in CA1 were induced by test stimuli at 0.05 Hz with an intensity that elicited a fEPSP amplitude of 40–50% of maximum. Test responses were recorded for 30–60 min before beginning the experiments to ensure stability of the response. To induce LTP, two consecutive trains (1 s) of stimuli at 100 Hz separated by 20 s. A protocol that induces LTP lasting ~1.5 h in wild-type mice of this genetic background were applied to the slices. The field potentials were amplified 100 × using Axon Instruments 700B amplifier (Foster City, CA, USA) and digitized with Digidata 1322A (Foster City, CA, USA). The data were sampled at 10 kHz and filtered at 2 kHz. Traces were obtained by pClamp 9.2 and analyzed using the Clampfit 9.2 (Molecular Devices, CA, USA).

### Statistical analysis

Statistical analysis of data was performed by the Dunnett's or LSD post-test based on the ANOVA for multivariate data analysis using SPSS 16.0 (SPSS Inc., Chicago, IL, USA). Data are expressed as means±S.E. of three independent experiments. Results were considered statistically significant at *P<0.05 (and **P<0.01).
