# Supplementary material for: Rapamycin upregulates glutamate transporter and IL-6 expression in astrocytes in a mouse model of Parkinson's disease
Source: Cell Death Dis. 2017 Feb 9;8(2):e2611–. doi: 10.1038/cddis.2016.491 (PMC5386462; doi:10.1038/cddis.2016.491)
Supplement: Supplementary Information [file cddis2016491x1.pdf]

SUPPLEMENTAL FIGURES

Figure S1

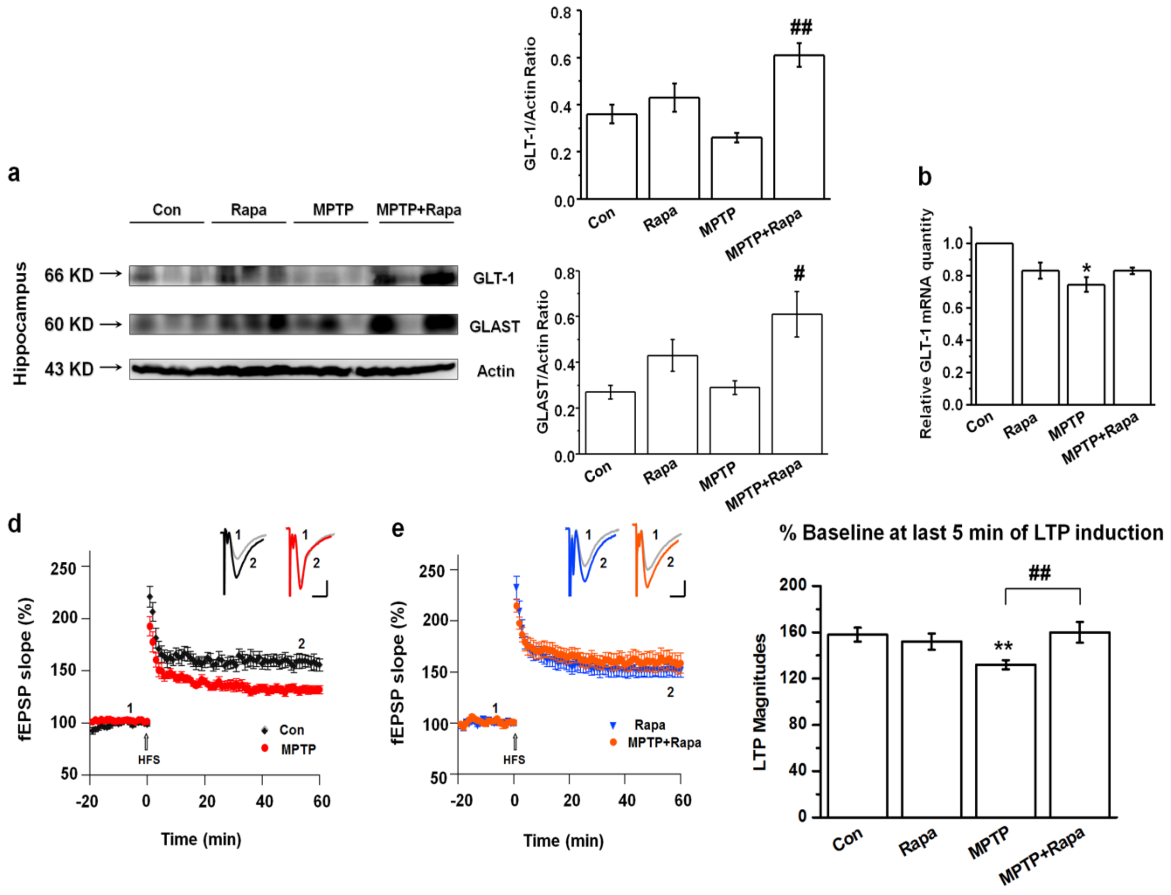

Figure S2

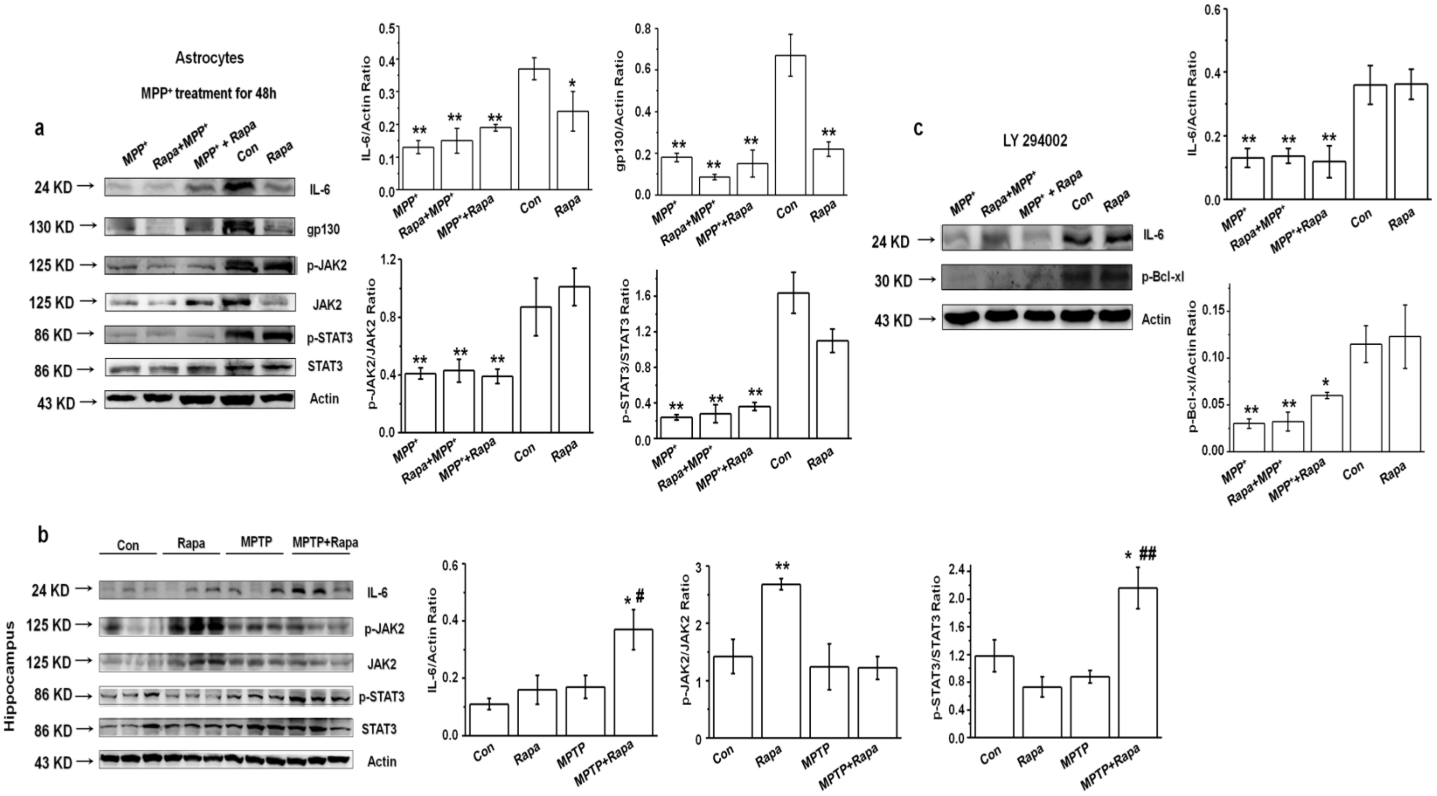

a
Astrocytes
MPP+ treatment for 48h
MPP+
Rapa+MPP+
MPP+ + Rapa
Con
Rapa
24 KD
IL-6
130 KD
gp130
125 KD
p-JAK2
125 KD
JAK2
86 KD
p-STAT3
86 KD
STAT3
43 KD
Actin
IL-6/Actin Ratio
gp130/Actin Ratio
p-JAK2/JAK2 Ratio
p-STAT3/STAT3 Ratio
c
LY 294002
30 KD
p-Bcl-xl
p-Bcl-xl/Actin Ratio
b
Hippocampus
MPTP
MPTP+Rapa

SUPPLEMENTAL FIGURE LEGENDS

Figure S1. Rapamycin prevents impairment of hippocampal synaptic plasticity by MPTP.

(a) The protein expression of GLT-1 and GLAST in the hippocampus upon application of rapamycin in MPTP-treated mice was determined by Western blotting (n = 3 per group, one-way ANOVA). **(b and c)** The mRNA expression of GLT-1 and GLAST in the hippocampus upon application of rapamycin in MPTP-treated mice was determined by qPCR assay (n = 3 per group, one-way ANOVA). **(d)** Compared to vehicle (black), the addition of 25 µM MPTP to the perfusion buffer for 50 min (red) does not change the baseline, but it inhibits LTP induced by high-frequency stimulation (HFS, arrow). Inset traces are typical field excitatory postsynaptic potentials (fEPSPs) recorded before (gray, as indicated at time point 1) and after (black or red, time point 2) HFS for each condition. Horizontal calibration bars: 10 ms; vertical bars: 0.5 mV. **(e)** Rapamycin (200 nM) added to the perfusion buffer for 60 min does not change the baseline and has no significant effect on the hippocampal LTP. Compared to the control (blue), pretreatment of rapamycin (orange) prevents MPTP impaired hippocampal LTP. Inset traces are typical fEPSPs recorded before (gray, as indicated at time point 1) and after (blue or orange, time point 2) HFS for each condition. Horizontal calibration bars: 10 ms; vertical bars: 0.5 mV. Each bar represents the mean ± SEM of at least three independent experiments. $^{*}p < 0.05$. $^{**}p < 0.01$, compared to the control group; $^{\#}p < 0.05$, $^{\#\#}p < 0.01$, for the MPTP versus MPTP+Rapa groups.

Figure S2. Effect of rapamycin on IL-6/JAK2/STAT3 expression in MPP^+-treated astrocytes and IL-6/JAK2/STAT3 expression in the hippocampus.

(a) Effects of rapamycin on IL-6/JAK2/STAT3 expression in MPP^+-treated astrocytes were examined by Western blotting. MPP^+ treatment was for 48 h (at least three experiments, one-way ANOVA). **(b)** Effects of rapamycin on the expression of IL-6/JAK2/STAT3 pathway proteins in the hippocampus in MPTP-treated mice were determined by Western blotting (n = 3 per group, one-way ANOVA). **(c)** Effects of the PI3K inhibitor LY294002 on the expression of IL-6 and p-Bcl-xl were determined by Western blotting (at least three experiments, one-way ANOVA). Each bar represents the mean ± SEM of at least three independent experiments. $^{*}p < 0.05$. $^{**}p < 0.01$, compared to the control group; $^{\#}p < 0.05$, $^{\#\#}p < 0.01$, for MPTP versus MPTP+Rapa groups.
